# Mass-Forming Ischemic Colitis: A Potential Mimicker of Malignancy

**DOI:** 10.1155/2019/8927872

**Published:** 2019-03-17

**Authors:** Alexandra M. Danakas, Bushra G. Fazili, Aaron R. Huber

**Affiliations:** ^1^Department of Pathology and Laboratory Medicine, University of Rochester Medical Center, 601 Elmwood Ave, Box 626, Rochester, NY 14642, USA; ^2^Department of Medicine Division of Gastroenterology/Hepatology, University of Rochester Medical Center, 601 Elmwood Avenue, Rochester, NY 14642, USA; ^3^Gastroenterology Group of Rochester, 2080 South Clinton Avenue, Rochester, NY 14618, USA

## Abstract

Ischemic colitis (IC) results from reduced colonic vascular perfusion, accounting for 50-60% of all gastrointestinal ischemic episodes. IC leads to mucosal damage with clinical symptom severity developing based on the duration and extent of colonic injury. In rare cases IC may form a mass-like lesion mimicking malignancy. Here we present the case of a 55-year-old female with hematochezia and diarrhea, who on workup was found to have a mass-like lesion at the ileocecal valve. Multiple biopsies demonstrated ischemic change and mucosal injury without evidence of dysplasia or carcinoma. Two months later on follow-up imaging, after supportive treatment the lesion was completely resolved. It is critical for gastroenterologists and pathologists to be aware of this variant of IC to avoid unnecessary surgical procedures and treatment of patients.

## 1. Introduction

Ischemic colitis (IC) is a common vascular disorder of the intestines, usually affecting older patients [1]. IC results from reduced vascular perfusion causing mucosal injury as the result of inadequate blood flow to the colon, leading to mucosal injury mediated by hypoxia followed by reperfusion. The pattern of injury is usually segmental, mainly involving the “watershed” zones of the splenic flexure, descending colon, and the rectosigmoid junction; however, any part of the colon can be affected. IC accounts for 50–60% of all gastrointestinal ischemic episodes, most often in the absence of major vessel occlusion [2, 3]. Clinically IC most commonly presents acutely with abdominal pain, hematochezia, and diarrhea, with more serious complications such as fever, perforation, peritonitis, and septic shock developing dependent on the duration and extent of colonic injury [4]. Often, the diagnosis can be made utilizing clinical, radiologic, colonoscopic, and mucosal biopsy findings; however, there have been reports of atypical pathologic manifestations, precluding the diagnosis [5]. The endoscopic appearance of transient ischemic colitis can vary including edematous and fragile mucosa; segmental erythema; scattered erosion; longitudinal ulcerations; petechial hemorrhages interspersed with pale areas; purple hemorrhagic nodules; worn vascular lakes; and sharply defined segment of involvement. In rare instances, IC may form a mass-like lesion, mimicking malignancy [3].

## 2. Case Presentation

**Figure 1:**
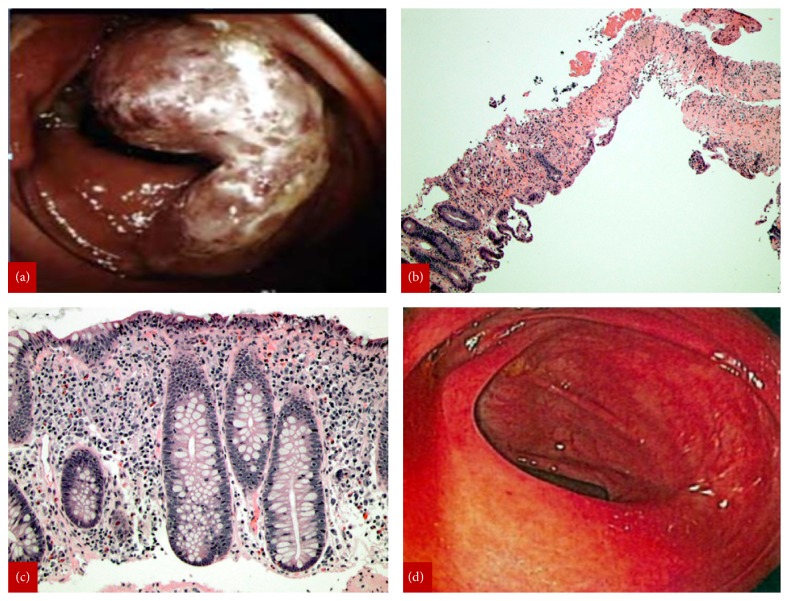
Initial colonoscopy findings showing mass-like lesion at the ileocecal valve (a). Biopsies of the ileocecal valve mass lesion demonstrating ischemic colitis ((b), original magnification x40). Background colonic biopsies demonstrating increase in intraepithelial lymphocytes, expansion of the lamina propria, and surface epithelial damage consistent with incidental lymphocytic colitis ((c), H&E original magnification x200). Follow-up colonoscopic image of ileocecal valve with complete resolution of the mass-like lesion (d).

A 55-year-old female with past medical history significant only for hypothyroidism presented to the ED with hematochezia and diarrhea of unknown etiology. Her last colonoscopy, three years prior, was unremarkable. On colonoscopy, a mass-like lesion was identified at the ileocecal valve (Figure 1(a)). Notably, on previous imaging studies, from three months prior, during a workup for painless jaundice, no mass was detected within the abdomen. Biopsies were taken of the mass-like lesion, as well as throughout the colon. The biopsies demonstrated hyalinized lamina propria, atrophic crypts, ulceration, and active inflammation, indicative of IC (Figure 1(b)). The random colon biopsies histologically demonstrated features of lymphocytic colitis (Figure 1(c)).

Three months prior to this ED visit she was treated with steroids for presumed autoimmune hepatitis following a workup and admission for painless jaundice, decreased appetite, right upper quadrant pain, “mustard yellow” urine and pale stools, elevated transaminases, and hyperbilirubinemia. An ultrasound was unrevealing, and CT showed no intrahepatic or extrahepatic bile duct dilatation, with a contracted gallbladder with pericholecystic edema of uncertain etiology. There was a nonspecific periportal edema extending towards the porta hepatis and a short segment jejunojejunal intussusception. Pathology of her liver biopsy showed an acute hepatitis pattern of injury with mild lobular cholestasis. The precise etiology was not readily apparent with the differential diagnosis including acute viral hepatitis and drug-induced liver injury. Plasma cells were inconspicuous, and the patient reportedly had negative F-actin serology; as a result, autoimmune hepatitis was not favored, though it could not be completely ruled out. The patient underwent a repeat colonoscopy two months later, following supportive treatment for her symptoms, although she endorsed additional episodes of diarrhea. The procedure revealed complete resolution of the mass-like lesion at the ileocecal valve with no signs of colitis (Figure 1(d)). After three months a CT angiogram was obtained which showed mild to moderate calcific atherosclerotic changes and mild tortuosity of the abdominal aorta without evidence of aneurysm or dissection. The celiac, superior, and inferior mesenteric arteries were widely patent without evidence of significant stenosis.

## 3. Discussion

Clues to the diagnosis of this clinicopathologic variant of IC include acute onset, rapidly changing course, elderly female patient, location in the right colon, ischemic colitis on histology after thorough sampling, and discordant imaging and colonoscopy findings [6]. Histologic evaluation exhibits lamina propria hyalinization and hemorrhage, a variable degree of epithelial damage in the form of mucin depletion, and crypt atrophy and loss, in addition to edema and fibrosis of the submucosa and muscularis propria. Of the few patients in the literature who have undergone colectomy, submucosal and/or mural edema or submucosal fibrosis were the only histologic findings. Therefore, adequate evaluation of the patients' clinical and histologic findings is instrumental to avoid a more extensive treatment [3, 5]. As in our case, these patients can usually be managed conservatively with resolution of the mass-like lesion on follow-up endoscopy. It is important for both pathologists and gastroenterologists to be aware of this variant of IC to avoid unnecessary surgery.
